# Global employability skills in the 21st century workplace: A semi-systematic literature review

**DOI:** 10.1016/j.heliyon.2023.e21023

**Published:** 2023-10-29

**Authors:** Hasanuzzaman Tushar, Nanta Sooraksa

**Affiliations:** a School of Human Resource Development, National Institute of Development Administration, Bangkok, Thailand; b College of Business Administration, IUBAT—International University of Business Agriculture and Technology, Dhaka, Bangladesh

**Keywords:** Employability skills, 21st-century workplace, Employers' expectations, SPAR-4-SLR

## Abstract

This study conducts a semi-systematic literature review of research pertaining to employability to identify essential employability skills that employers seek in recent graduates. The comprehensive analysis of the existing literature review aims to present a set of global employability skills, identify similarities, variations, or changes in these skills across time, and explore the most relevant existing employability skills for the 21st-century workplace. The review includes 30 years of research articles and government reports published in English and considers 25 studies based on the Scientific Procedures and Rationales for Systematic Literature Reviews (SPAR-4-SLR). After removing duplicates, 87 unique skills were identified and listed under three distinct temporal themes (the 1990s, 2000s, and 2010s), with problem-solving, communication, teamwork, adaptability, and willingness to learn among the most commonly reported skills over time. The study found a mismatch between employers' expectations and graduates' possessed skills. Therefore, the list of employability skills identified in this study can serve as a valuable tool for addressing this mismatch. The study's findings can also help educators and employers to better align their efforts to prepare students for the modern workplace.

## 1 Introduction

The 21st century has significantly changed the way of work and the workplace environment. Advancements in technology, socioeconomics, and industry have greatly affected organizations. These changes are anticipated to further intensify in the future, especially in the aftermath of the COVID-19 pandemic [1]. In order to adapt to this rapidly evolving landscape, organizations must have employees who are dedicated to excelling in technical and professional skills, embracing emerging technologies, demonstrating self-motivation, and actively engaging in their work. These are essential for achieving high levels of competence, confidence, and the ability to complete tasks effectively [2,3]. It is broadly termed employability skills that are recognized as a constructive approach that can help to meet today's challenges in the modern workplace. However, the majority of research studies consistently indicated that graduates continue to lack market-driven employability skills necessary for success in the 21st-century workplace [[4], [5], [6], [7], [8]].

As per the IMF [9], there is an ongoing increase in global economic growth (forecasted at 5.5 % growth in 2021 and 4.2 % in 2022). Nonetheless, the challenge of unemployment is concurrently surging significantly in developing and less developed nations. As stated by the ILO [9], the global count of unemployed individuals reached around 201 million in 2021, marking a slight increase from the previous year. However, it's worth noting that this number is likely to have been affected by the COVID-19 pandemic, which has significantly impacted the labor market and the global economy. In recent years, the lack of job opportunities for the young working-age population has been an emerging challenge for many regions, i.e., Northern Africa (30 % youth unemployment rate in 2021), Arab states (27 %), and Southern Asia (13 %).

Based on a survey conducted by Manpower Group, a leading workforce solutions company, in 2020, around 42 % of employers globally faced challenges in recruiting skilled employees. The Forbes Human Resources Council [9] expresses concern over the most significant challenge being the skills gap within companies, a challenge that is projected to become even more profound. Employers are similarly concerned about addressing skill gaps through various strategies, including hiring an employee with possessed skills for the modern workplace, retraining the existing employees, and using automation technology. Employers also consider that higher educational institutions are responsible for developing such required skills, as reported by some studies (e.g. Refs. [[10], [11], [12], [13]]). Moreover, Qenani et al. [13] posited that universities ought to serve as source of culture and creativity, fostering the creation of knowledge, traits, and skills essential for both students’ personal growth and their professional lives. However, numerous empirical works continuously reported that universities' academic programs are outdated and fail to fulfill the labor market expectations [12,[14], [15], [16], [17]]. This current mismatch is observed by many studies all over the world, e.g., Australia [18], the USA [19], the U.K. [20], China [21], South Africa [22], Vietnam [23], Spain [3], Malaysia [24], India [25,26], and Bangladesh [27].

While numerous studies have delved into the topic of employability skills, this paper aims to provide a fresh perspective by addressing specific gaps in the existing literature. The primary rationale behind this study is to shed light on the growing concern that recent graduates often lack the market-driven employability skills demanded by the modern workplace [6,7,28]. This discrepancy has far-reaching implications for the labor market, as evidenced by the simultaneous rise in global unemployment rates, particularly in developing and less developed countries [9,29].

The implications of this gap in employability skills extend beyond academia and directly impact graduates' employability prospects and long-term career trajectories. The disconnect between what universities impart and what the labor market demands underscores the urgency to bridge this gap. Failure to address this issue could lead to prolonged periods of unemployment, reduced workforce productivity, and economic stagnation, impacting both regional and global contexts. Consequently, there is a compelling need for comprehensive research to explore and understand the intricacies of this mismatch and propose viable solutions to align employability skills with current and future workplace demands.

Numerous extant literature review studies on employability skills have been conducted with varying purposes and across different timeframes. For instance, Sarfraz, Rajendran, Hewege, and Mohan [30] categorized employability skills from diverse stakeholder perspectives. Williams, Dodd, Steele, and Randall [31] explored to conceptualize the notion of employability, while Neroorkar [32] scrutinized the variations of employability skill measures. Additionally, Eldeen, Abumalloh, George, and Aldossary [28] identified industry-specific skills. However, this study aims to explore, synthesize, and analyze literature on employability skills for fresh graduates, conducted in a 30-year period from 1990 to 2019 through a semi-systematic review. Subsequently, the study seeks to provide a comprehensive list of required employability skills for fresh graduates, perceive similarities, differences, or changes in these skills over time, and examine the key employability skills that are most valued in today's 21st-century workplace.

### 1.1 Research questions and objectives

The above-discussed problem statement underscores an apparent mismatch between employers' expectations and graduates' possessed skills across the globe. Therefore, obvious questions may arise regarding employers' expectations from fresh graduates and how to get informed about the students’ employability skills. These fundamental questions led the researcher to identify employability skills through a semi-systematic literature review. The questions employed to navigate this study are as follows.

- What are the essential employability skills employers want from fresh graduates?
- What are the variations and shifts in employability skills over the specified period?

## 2 Methods

The current study carried out a thorough examination of the literature on employability by conducting a semi-systematic literature review. Literature reviews are crucial for analyzing and bringing together different studies [33]. According to Snyder [34], a semi-systematic review typically examines the progression of research within a chosen field over time or traces the development of a particular topic across various research traditions. Considering the diverse spectrum of disciplines that underpin the literature on employability skills, conducting a fully systematic and quantitative meta-analysis was unfeasible. Consequently, a semi-systematic method was employed, enabling the derivation of content or thematic analysis from the identified seminal literature [35].

**Fig. 1:**
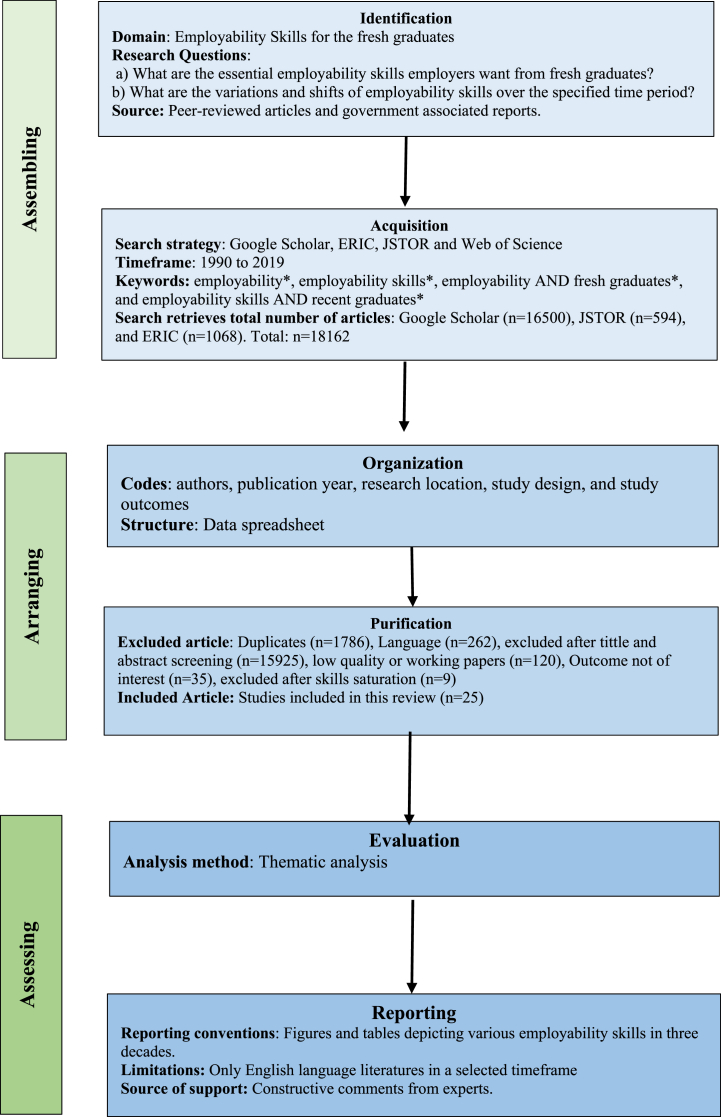
The Flowchart of SPAR‐4‐SLR protocol.

This study followed the SPAR-4-SLR protocol as described by Ref. [36]Paul et al. [36] to ensure the transparency of the literature review procedure. While PRISMA and PRISMA-P are more descriptive in nature, they might have limited applicability for reviews seeking to contribute theoretically [36]. Assembling, arranging, and assessing are the three main phases of the SPAR-4 SLR process. Each of these phases has six sub stages, including identification, acquisition, organization, purification, evaluation, and reporting [36]. Fig. 1 presents a schematic outlining the methodology, which is subsequently elaborated upon in the following section.

At the initial phase (assembling), this study identified peer-reviewed and government-associated published literature by employing a thorough and refined search strategy. The following three online databases were used in the search for relevant articles: Google Scholar, ERIC, and JSTOR. Further citations were obtained by reviewing the reference lists of relevant literature related to employability skills. The following keywords were used to define search criteria and to identify employability skills: “employability*“, “employability skills*“, “employability AND fresh graduates*“, and “employability skills AND recent graduates*“. The search strategy was limited to full-text literatures in English published between 1990 and 2019.

This study conducted searches for relevant literatures that explicitly incorporated the selected terms in the title, abstract, keywords and/or full contents. The search keywords were consistent across all databases. In this initial phase, research studies with titles referencing employability skills (such as models, measurement scales, or tools) were included, and their abstracts and/or keywords were reviewed. In cases where the title alone did not adequately establish relevance to the inclusion criteria, the complete manuscripts were accessed, and their whole contents were retrieved for detailed examination to determine if they met the inclusion requirements. The current study kept the literatures that addressed three inclusion criteria: a) articles in English bearing titles mentioning employability skills, b) articles published between 1990 and 2019, and c) concerned employability skills required for fresh graduates. The search process retrieved a total of 18,162 studies: 16,500 studies in Google Scholar, 1068 in ERIC, and 594 in JSTOR. To find the studies for reviews, various word combinations were employed.

In the second phase (arranging), each piece of literature underwent a thorough examination using the data extraction sheet after obtaining the list of manuscripts that fulfilled the initial inclusion criteria. A data spreadsheet was generated to record the subsequent organizational codes extracted from the included studies: authors, publication year, research location, study design, and study outcomes. During the purification stage, a total of 1786 duplicated literature sources were disregarded, leaving an additional 15,925 for elimination after evaluating titles and abstracts. Further refinement led to the exclusion of 262 articles due to non-English language publications, and 120 studies from low-quality sources and working papers were also omitted from the analysis. A total of 35 studies were discarded as their outcomes and key content were found to be less relevant and not aligned with the inclusion criteria. Following this rigorous process, 34 studies were obtained from the literature search and their full content was retrieved for further examination. As the aim of this study was to compile a comprehensive global set of employability skills, a process of skill saturation was applied to identify unique skills, resulting in the exclusion of 9 studies. Ultimately, the current review encompassed 25 seminal journal articles and government-associated reports. To ensure reliability, the co-author also reviewed the extracted pieces of literature. In addition, a reviewer not associated with the study assessed the quality of the data.

In the last phase (assessing), the categorization of employability skills for this review employed a thematic analysis approach. A total of 87 skills were identified across 25 seminal literatures. Unifying themes within these skills were identified and subsequently classified into three distinct temporal dimensions: the 1990s, 2000s, and 2010s. This study also formulated a roadmap for future research endeavors. In the concluding sub-stage (reporting), the study utilized tables and figures to identify, synthesize and analyze the variation or shifts of employability skills across different periods. Furthermore, the study evaluated the limitations and the pragmatic implications of the outcomes.

## 3 Findings

The notion of employability was relatively vague two decades ago and has now permeated workplaces, governmental policies, and literature in multiple disciplines (i.e., psychology, education, sociology, management, policy studies, and career research) over the past two decades [37]. The usage is somehow connected to all the recent changes in the exponential work and workplaces, i.e., social, economic, demographical, and technological. The recent push from the challenging labor market and changing global environment promotes the notion of employability and its implications. In many countries, it takes a central place in the national labor and higher education policies for the welfare of large cohorts of young labor market entrants ILO. [38]. Notably, it has become one of the four pillars of the European employment strategy [37,39] and a significant national policy on youth employment in the United Nations. Thereafter, many terms and sets of skills are formed and used in the literature to refer to employability skills, i.e., attributes, competencies, qualities, professional skills, generic skills, and characteristics [8]. However, this review process attempts to generate a set of general graduate employability skills and perception of employers’ expectations from fresh graduates.

To generate a primary pool of skills, the following three sections discuss the most cited reports and studies from an extensive literature review from 1990 to 2019. The three temporal dimensions attempted to provide a comprehensive overview of employability skills in the historical period (i.e., the 1990s, 2000s, and 2010s). The study selected 25 significant reports and research studies to provide a synopsis and to look at the variation or shift of employability skills over time. The selected five studies were published in the 1990s, six in the 2000s, and the other fourteen in the 2010s.

### 3.1 1990s

During the 1990s, a number of noteworthy reports and studies (see, for example [[40], [41], [42], [43], [44]]) explained the required skills for the employers’ exponential needs of the world of work. Notably, two reports originating from the USA [44,45] are often cited in contemporary employability literature as foundational works to identify the skills. The American Society for Training and Development (ASTD) conducted the study in 1990 with the support of a U.S. Department of Labor grant [46]. It published the work titled “*Workplace basics: The essential skills employers want.”* The study found 16 skills categorized under six dimensions, including “fundamental skills-literacy, writing, and arithmetic”; “communication-verbal, auditory”; “adaptability-problem solving, creative thinking”; “self-improvement - career planning, motivation, self-esteem, and goal setting”; “group effectiveness skills- teamwork, social skills, and negotiation”; and “influencing skills-accepting organizational culture, and leadership” [45]. The ASTD study emphasizes the perspectives of employers and lists workplace basic skills at all levels of workers.

Afterward, the U.S. Department of Labor established the Secretary's Commission on Achieving Necessary Skills (SCANS) in 1991 to update the Carnevale et al. [45] report. The SCANS report revised the skills through the perspectives of both employers and graduates. The study was concluded by proposing 36 essential skills categorized into three foundational skill sets and five competencies. The five competencies include resource management (selecting, organizing, allocating time, money, staff, resources, and space), interpersonal skills (teamwork, negotiating, and leading, serving, or teaching others), information (acquiring, using, evaluating, interpreting and communicating information properly), understanding system (analyze social, organizational, and technological structures to observe and improve its performance), and technology (selecting, applying, maintaining, and troubleshooting technologies). The three components of the skills are: “fundamental abilities (literacy, writing, arithmetic, auditory, and verbal skills)," “cognitive abilities (innovation, willingness to learn, solving problems, and reasoning)," and “personal attributes (accountability, self-worth, self-control, social competence, and ethics)." The scope and implication of the SCANS report are much broader than the ASTD report in identifying young people's employability skills. Despite this, both studies are examples of great insight into employability skills and have been acknowledged by industry and higher education to date.

In a study within the same decade, McLaughlin [42] developed an evolving employability skills profile essential for Canadian employers, with support from the Conference Board of Canada. The objective was to prepare students for the future workplace. The study mainly emphasized the perception of the multiple stakeholders. The findings outlined all essential skills under three dimensions: academic skills (able to communicate, think, learn, solve problems, use technology and subjective knowledge), self-management skills (demonstrating positive attitudes and behaviors, self-esteem, integrity, and personal ethics, creativity and initiative, ready to take responsibility and adaptability toward change), and teamwork skills (able to work with others, understand others culture, plan and make decisions with team, lead and organize the group for high performance). The Association of Graduates Recruiters [40] added some individual-centric skill sets for U.K. graduates in the same year. The skills include self-awareness, self-esteem, action planning, self-promotion, and networking.

Moreover, Kajihara [47] developed 22 employability qualities required by Japanese employers. The study was based on a survey of 200 Japanese companies supported by the Ministry of Labor. The proposed skills include vitality, honesty, responsibility, adaptability, personality, sociability, dedication, ambition, initiative and creativity, ready-to-face challenges, and visionary thinking. The study also found that the required employability skills vary from industry to industry (i.e., manufacturing prefers honesty and sociability on top; financial organizations value more on enterprise, honesty, responsibility, and adaptability; engineering organizations appreciate creativity above all) [48].

Unlike the different sets of skills, there are some common agreement between the studies mentioned above. After careful analysis of the selected studies and government-associated reports from other countries between 1990 and 1999, 85 skills were found in total, whereas 39 unique skills were identified after removing the duplicate skills (see Appendix 1). Table 1 presents the common top ten employability skills in this decade, including problem-solving, self-esteem, teamwork, communication, creativity and initiative, interpersonal skills, adaptability, responsibility, goal-setting, and learning. All the studies in this period reported that problem-solving, self-esteem, and teamwork are the utmost essential skills expected by employers. Communication is the next commonly reported skill, followed by creativity and initiative. Apart from the top ten skills, there are also some widely reported skills, including planning and decision-making, critical thinking, basic IT skills, leadership, integrity, and personal management.

**Table 1:** Common top ten employability skills in the 1990s.

Employability Skill (1990–1999)		Problem solving	Self-esteem	Teamwork	Communication	Creativity and initiative	Interpersonal skills	Adaptability	Responsibility	Goal-setting	Learning
Studies/Report	Context										
ASTD [46]	USA	X	X	X	X	X	X	X		X	
SCAN [44]	USA	X	X	X	X	X	X		X		X
McLaughlin [42]	Canada	X	X	X	X	X	X	X	X		X
AGR [40]	UK	X	X	X	X					X	
Kajihara [47]	Japan	X	X	X		X		X	X	X	X

### 3.2 2000s

Secondly, during the 2000s, numerous significant studies (refer to Table 2) took place on the concept of employability skills. This study selected the literature from diverse countries to observe the variation of the skills needed by employers from fresh graduates. In 2002, the ACCI-Australian Chamber of Commerce and Industry, associated with the BCA-Business Council of Australia (BCA) and the Department of Education, Science and Training (DEST), published a research project titled “*Employability Skills for the Future”* [49]. They proposed an employability skills framework with a large set of facets of the skills under eight essential skills (communication, problem-solving, teamwork, initiative, and enterprise, personal management, planning and organizing, learning, and technology) in conjunction with the personal competencies (loyalty, commitment, adaptability, integrity, self-esteem and so on). Later, in 2006, the Government Skills Australia (GSA), operating under the Department of Education, also promoted the aforementioned skills framework to prepare graduates for employment and future learning.

**Table 2:** Common top ten employability skills in the 2000s.

Employability Skill (2000–2009)		Communication	Team work	ICT skill	Problem solving	Self-esteem	Creativity and initiative	Self-management	Planning and organizing	Adaptability	Time Management
Studies/Report	Context										
ACCI [50]	Aus.	X	X	X	X	X	X	X	X	X	X
Bennett [51]	UK	X	X	X	X	X	X		X	X	
CBI [52]	UK	X	X	X	X		X	X		X	X
Andrews & Higson [53]	EU	X	X	X	X	X	X	X	X		X
Archer, & Davison [54],	UK	X	X			X			X		
Bridgstock [55]	Aus	X	X	X	X	X	X	X		X	X

In the UK, Bennett [51] researched 1000 job advertisements to outline the transferable skills required by employers. The study found that employers' top requirements are communication, I.T., organization, teamwork, interpersonal, motivation, analytical, and self-esteem. CBI [52] suggested that the fundamental skill for the U.K. graduate is a positive attitude. The report underpinned seven other competencies: self-management, problem-solving, ability to work in a team, application of numeracy and I.T., business and customer awareness, communication, and literacy. The report also revealed that employers are dissatisfied with the following skills of new graduate recruits: foreign language skills, business awareness, and self-management, information about the job or career, and positive attitude toward work. Furthermore, Archer and Davison [54] added a few more on the satisfaction gaps in the skills of new graduates: decision-making skills, good writing skills, communication, experience related to a potential career, confidence, passion, and personal development skills. On the other hand, the report also considered that employers are most satisfied with I.T. skills, academic qualifications and skills, and intellectual ability.

During the same period, Andrews and Higson [53] conducted research across four European Union countries (UK, Austria, Slovenia and Romania). Their study identified the critical skills required by the employers, considering perspectives from both graduates and employers. The study suggested that employers prefer a graduate with interpersonal competencies, complex business-related knowledge and application, and work experience through a work-based learning program (such as internship or job placement) above all. The study also conceptualized the key transferable graduate employability skills through synthesizing the literature, including professionalism, reliability, adaptability, workability under pressure, networking, teamwork, verbal and written communication skills, ICT skills, creativity, self-esteem, self-management, time management, and willingness to learn. To elaborate further, Bridgstock [55] proposed a notional model of graduate employability skills with a few essential components, i.e., career-building skills, personal management skills, generic skills, subject-specific skills, and underpinning personalities and dispositions. The study pointed out that academic skills, communication, information and technology literacy, career-building, and management skills are essential for a graduate to build a career.

Considering the aforementioned literature review between 2000 and 2009, a total of 123 skills were found, and 59 unique skills were synthesized after eliminating the duplicate skills (see details in Appendix 1). The common top-ten skills are shown in Table 2, including communication (reported in all the selected studies), teamwork (reported in all the selected studies), ICT skills (reported in 5 studies), problem-solving (reported in 5 studies), self-esteem/confidence (reported in 5 studies), creativity and initiative (reported in 5 studies), self-management (in 4 studies), planning and organizing (in 4 studies), adaptability (in 4 studies), and time management (in 4 studies). Besides the top-ten skills, other commonly reported essential employability skills include integrity, responsibility, critical thinking, willingness to learn, motivation, networking, passion, attitude, negotiating, commercial awareness, and name a few.

### 3.3 2010s

Thirdly, from 2010 to 2019, it is documented that a significant number of studies focused on employability skills in various contexts. Notably, attention has continuously increased in the recent years. This study selected fourteen studies in different contexts to provide a synopsis of the required employability skills of fresh graduates. Rosenberg, Heimler, and Morote [56] outlined eight dimensions of basic employability skills with 47 items, such as basic numeracy skill and literacy, critical thinking skill, problem-solving, communication, teamwork, adaptability, interpersonal skill, management skill, leadership skill, application of I.T. skill, system thinking skill, and work ethic. More recently, Bloomberg [57] suggested the top 5 soft skills required by employers in the U.S., including teamwork, critical thinking, complex problem-solving, adaptability, and ethical judgment.

Humburg and van der Velden [58] considered six types of skills, namely professional skills, innovative and creative, interpersonal, general academic skills, strategic and organizational skills, and commercial and entrepreneurial skills. Their study highlighted the significance of these skills for the graduate recruitment process across European countries. In Greek, Matsouka and Mihaly [59] suggested that Greek companies value more on a fresh graduate with the following skills: learning orientation, teamwork, extra effort, integrity, communication, professionalism, adaptability, goal setting, emotional intelligence, motivation, self-awareness, and initiative and creativity. The study also reported that the utmost mismatch skills are goal setting, influential skills, leadership, self-awareness, professionalism, and emotional intelligence.

The research on employability has received less attention in developing and least-developed countries, particularly within the South Asian region. However, in recent years, a few studies have emerged as a result of an extensive literature search process (refer to Table 3). Srivastava and Khare [60] conducted qualitative research in the Indo-centric region (i.e., India, Pakistan and Bangladesh) and focused on employers’ perceptions of the required employability skills. The study suggested that critical thinking, willingness to learn, leadership, communication, analytical skills, time management, hardworking, positive thinking and attitude, and problem-solving were valued by Indian employers. Blom and Saeki [61] listed 26 skills and categorized them into three factors based on the importance ratings by Indian employers while recruiting fresh engineering graduates. The three factors include core employability skills (integrity, entrepreneurial skills, self-discipline, willingness to learn, flexibility, empathy, and teamwork), professional skills (problem-solving, customer service skills, application of academic knowledge, and creativity), and communication skills (written and verbal communication, English language communication, ICT skills, technical skills, and few others). To this list, the Economist [62] added English language communication, quantitative skills, and advanced-level computer skills were also essential in India. More recently, the India Skill Report (ISR) [63] ranked communication skills, adapting skills to the changing environment, and willingness to learn are the top preferences of Indian employers above all. The report also indicated that interpersonal skills, learning agility, conflict resolution, emotional intelligence, and self-determination are crucial for the Indian job market.

**Table 3:** Common top ten employability skills in the 2010s.

Employability Skill (2010- latest years)		Communication	Problem solving	Adaptability	Team work	Analytical/critical thinking	Willingness to learn	Integrity	Interpersonal	ICT Skill	Leadership
Studies/Report	Context										
Blom and Saeki [61]	India	X	X	X	X		X	X		X	
Srivastava & Khare [60]	South Asia	X		X	X	X	X	X	X	X	X
Rosenberg et al. [56]	US	X	X	X	X	X	X	X	X	X	X
Economist [62]	South Asia	X	X	X						X	
Mirza, Jaffri, & Hashmi [64]	Pakistan	X	X		X			X	X	X	
Su & Zhang [21]	China	X	X		X	X	X	X	X		X
Humburg & van der Velden [58]	EU	X	X		X	X			X		
Yang, Cheung, & Fang [65]	China		X	X		X	X	X			X
Matsouka & Mihaly [59]	Greek	X		X	X		X	X		X	X
Chowdhury and Miah [27]	Bangladesh	X	X	X	X	X	X	X	X		X
Bloomberg [57]	US		X	X	X	X		X			
Nghia [23]	Vietnam	X	X	X	X	X	X		X	X	X
WEF [66]	General		X			X	X			X	X
ISR [63]	India	X	X	X			X		X		

Pakistani employers prefer voluntarism, leadership, communication, entrepreneurial skills, career planning, loyalty, conceptual understanding, and respect for elders [60]. To add in this list, Mirza, Jaffri, and Hashmi [64] identified 24 essential skills under three categories for Pakistani employers when hiring new graduates. The categories were based on the employers' perception that includes communication and business-specific skills (verbal communication, customer service skills, interpersonal skills, entrepreneurship, problem-solving, proper planning, and organizing), core employability skills (teamwork, hard work, self-discipline, self-motivation, initiative), and professional skills (decision-making skill, application of academic knowledge, ability to use of modern tools, ICT skills, integrity, self-awareness, and technical skills). The study's findings identified the gap in possessed professional skills among Pakistani students and employers are also least satisfied with it.

Building upon the findings of Srivastava and Khare (2012), their research revealed that employers in Bangladesh expect a range of skills from their workforce. These include attributes such as hardworking, interpersonal, behavioral, adaptability, respect for labor, language proficiency, safety awareness, ICT proficiency, business acumen, commitment, sincerity, negotiation skills, and familiarity with the Kaizen method. Recent studies by Chowdhury and Miah [67] posited a seven-dimension with 30 employability skills for entry-level H.R. positions in Bangladesh. The dimensions were derived from the perspectives of both students and employers, encompassing personal skills (confidence, team building, self-awareness, well-groomed, listening, organizing, and enjoying challenges), specific communication skills (applying knowledge, info management, breadth knowledge, written communication, subject knowledge, work ethic, and safety), academic skill (relevant internship, taking major courses, and activity-based intern), integrity skill (commitment, honesty, attitude, and responsibility), generic skill (critical thinking, problem-solving, judgment ability, negotiation, and creativity), interpersonal (networking, up to date knowledge, and media communication), adaptability (professionalism, self-management, and flexibility), and students valued differently on these two skills influential (university image, type of university, and reference), and team skill (team leading, compromiser, encourager, and evaluator).

In China, Su and Zhang [21] proposed a competency model with 16 employability skills under five dimensions based on both perspectives of students and employers. The skills include responsibility, teamwork, professional knowledge, moral quality, creativity and initiative, problem-solving, and communication. In addition, Yang, Cheung, and Fang [65] proposed 15 measurement items of employability skills for entry-level hotel employees in China. The 15 significant skill items were grouped into four skill dimensions, including job performance and self-management (adaptability, integrity, working independently, enthusiasm, and positive attitude), organization and time management (responsibility, setting priorities, and time management), creativity and innovation (willingness to learn, innovative, creative and initiative), and problem-solving skills (leadership, identifying problem and solution, analytical and critical thinking). On the other hand, Nghia [68] specified 35 skills with six dimensions that are sought by the employers in Vietnam. The six dimensions include career development skills (decision-making, career planning, planning and organizing, adaptability, self-awareness, management, and leadership skill), learning and personal development skills (willingness to learn, responsibility, problem-solving, teamwork, and self-assessment), interpersonal and communication skills (sociability, foreign language, presentation, self-esteem), intellectual skills (creativity, basic numeracy, and critical thinking), literacy skills (reading, writing, and listening skills), and information skills (information management, ICT skills, learning resources searching skills).

More recently, WEF [66] proposed a top ten comparing skills expected by employers over the 2018 to 2024 period. The report posits that analytical and critical thinking, complex problem solving, active learning, creativity and initiative, trustworthiness, emotional intelligence, reasoning, time management, leadership, and social influence are essential skills. To this list, system analysis, evaluation, technology design, and programming will be added as trending skills in 2024. On the other hand, manual skills and physical abilities, basic skills (reading, writing, math, and listening), technology installation and maintenance, and monitor and control skills declined in 2022. The India Skill Report (ISR) [63] ranked communication skills, adapting skills to the changing environment, and willingness to learn are the top preferences of Indian employers above other skills. The report also indicated that interpersonal skills, learning agility, conflict resolution, emotional intelligence, and self-determination are essential for the Indian job market.

Taking into account all the discussed studies between 2010 and 2019, a cumulative total 237 skills were identified. After eliminating the duplicated skills, 69 unique skills were specified (see Appendix 1). Table 3 indicates the commonly reported top-ten skills are as follows: problem-solving (appeared in 12 studies), communication (appeared in 11 studies), adaptability (in 10 studies), teamwork (in 10 studies), analytical and critical thinking (in 9 studies), ready to learn (in 10 studies), ethics and integrity (in 9 studies), interpersonal (in 8 studies), leadership (in 8 studies), and ICT skills (in 8 studies). Despite the top ten skills, other commonly demanded skills include creativity and initiative, positive attitude, hard work, professionalism, emotional intelligence, time management, appearance, responsibility, and so on.

## 4 Discussion

The analysis encompassed research studies and government-related reports published in English over the span of the past 30 years. In the initial stage, 38 unique skills were reported in the 1990s, 59 in the 2000s, and 69 in the 2010s. In total, 166 skills were found, whereas 87 unique skills were sorted after eliminating the duplicate skills. However, few skills were industry-specific (e.g., accounting knowledge, system analysis, programming, and so on), 33 skills appeared in one study, and 10 appeared in only two studies. The commonly reported top 15 employability skills (1990–2019) are as follows: problem-solving (in 22 studies), communication (reported in 21 studies), teamwork (in 21 studies), adaptability (in 17 studies), willingness to learn (in 17 studies), creativity and initiative (in 17 studies), ICT skill (in 16 studies), analytical/critical thinking (in 15 studies), integrity (in 15 studies), interpersonal skill (in 14 studies), self-esteem (in 13 studies), leadership (in 12 studies), planning and organizing (in 12 studies), responsibility (in 11 studies), and self-management (in 10 studies).

The purpose of this semi-systematic literature review was two folds: first, to see the similarity, variation, or shift of employability skills over the selected time, and second, to explore and list the existing employability skills required by employers from fresh graduates that served the research question of this study. The literature review findings revealed that communication, problem-solving, and teamwork remained the top required employability skills in the selected period. Notably, computer and technology-related skills are valued more in recent literature due to the new wave of automation technology. Besides time management, emotional intelligence, work ethics, judgment ability, entrepreneurial skill, hardworking, respecting seniors, foreign language, and practical experience are only reported in the 21st century studies. Some skills (i.e., hardworking, respecting seniors, and entrepreneurial skills) are reported only in South Asian literature, and some skills (i.e., work ethics, judgment ability, and emotional intelligence) are identified in the 2010s literature.

Despite the similarities and variations in employability skills, the literature review also indicated a shift in these skills over time. The earlier studies reported that basic literacy, numeracy, manual skills, and skills related to financial management were on top of the list of employability skills. On the other hand, the recent employability skill report (see, example [66,69]) indicates that these skills are continuously declining. Expectedly, Tymon [8] stated that “the increase in the number of graduates has also changed employer's expectations” (p. 848). In addition, Suarta et al. [69] reported that employers are not satisfied enough with graduates possessing occupation-specific skills in the 21st-century labor market. Some required skills by employers include emotional intelligence, leadership and integrity, critical thinking, adaptability, and creativity and innovation. These skills are also well-documented in recent studies, e.g. Refs. [59,63,67,66], with an ongoing surge in demand. Notably, employability skills exhibit variations across countries, industries, and roles/positions and even within stakeholder groups [8].

The study also found a prevalent mismatch between employers' expectations and graduates' possessed skills, a phenomenon not unique but widespread across many nations. This highlights the need for employers, educators, and policymakers to work together to ensure that graduates have the necessary skills to meet the demands of the modern workplace. Overall, this study provides valuable insights into the employability skills most in need in the 21st-century workplace across the globe and can help employers, educators, and policymakers better prepare young people for success in the modern workforce.

## 5 Theoretical and practical implications

Employability is a field that remains a greater interest in higher education and academic literature in this century [70]. Several studies have taken place in the last few decades to scrutinize the importance of higher education in developing societies and the economy. The value and effectiveness of higher education are primarily demonstrated through two main sources: governments and employers [12]. As a result, employers hold high expectations from universities and graduates.

The implications of this study are significant for educators, employers, and policymakers across the globe. The study's findings can help educators better align their curriculum and teaching methods with the employability skills employers are looking for in fresh graduates. By identifying the specific skills that are most in demand, educators can ensure that their students are well-prepared for the modern workplace and have the best chance of finding employment after graduation. Additionally, employers can use the study's findings to reshape their recruitment and hiring processes and design training programs that target the skills most important for success in the 21st-century workplace.

The study's findings can also inform policymakers as they design policies and programs to support workforce development. The government can use the study's findings to guide the development of programs that promote employability and career readiness among young people and to support the development of industries that require the skills identified in the study. Moreover, the study's results can be used by students, potential job seekers, and career counselors to identify the skills they need to acquire to increase their employability and career readiness. It can help them focus on the skills employers are looking for and make informed decisions about further education and training. Educators and decision-makers can use the specified collection of skills to design curriculum, global employability initiatives, and related legislation to prepare graduates for the global workforce.

## 6 Conclusion and future research directions

The semi-systematic literature review provides a set of global employability skills categorized into three distinct decades: the 1990s, the 2000s, and the 2010s. It outlines similarity, variance, and a unique set of skills in this technological advancement era. The literature review also discussed a historical overview and several perspectives to define employability skills. This information would be useful to current students, academic institutions, potential employers, and policymakers. The study also suggests that creating an employability scale based on employers' expectations can serve as a valuable tool for identifying and addressing the mismatch between employers' expectations and graduates' possessed skills.

The impact of the global predicament will be experienced more by the rising economies as the working population of the globe is anticipated to rise at the same time that employment prospects are likely to decline [29]. Therefore, students and fresh graduates must prepare themselves with the necessary skills and competencies in addition to their degrees to succeed in a competitive workforce. Academia and organizations must work together to ensure fresh graduates possess the essential skills. Educational policymakers should concentrate on developing curricula and modernizing instructional strategies based on organizational expectations. Delving into industry-specific employability skill needs could provide targeted insights for educators and policymakers, enabling them to align curricula and teaching initiatives more effectively. On the other hand, business organizations ought to assist academic institutions in equipping graduates for the workforce, who will one day become the backbone of their respective industries. Further research should conduct longitudinal studies to track the evolution of employability skills over extended periods that could offer a deeper understanding of how these skills continue to adapt to changing workplace dynamics.

In addition, the study primarily relies on the examination of employability skills over a 30-year period published in English, while illuminating evolving trends, might exclude valuable perspectives from non-English sources and recent developments. Therefore, exploring regional variations in employability skills demands and offerings could shed light on the unique requirements faced by diverse geographical regions, helping tailor educational and training programs accordingly. Comparative analysis across cultures and nations may uncover cross-cultural variations in employability skills preferences and their implications, aiding in the development of globally applicable strategies. Certainly, addressing the evolving landscape of employability skills in the wake of the COVID-19 pandemic and the future of work is a critical aspect of future research in this field. Recent global events have significantly accelerated the adoption of remote work, digital technologies, and automation [71]. These shifts are likely to have profound implications for the skills that employers seek in fresh graduates. Research should, therefore, endeavor to connect the findings of this study with the emerging trends in the post-pandemic workforce. Exploring the impact of emerging technologies, as supported by recent studies [[72], [73], [74]], on the demand for employability skills and how they are reshaping skill priorities is an area of significant relevance. By addressing these areas, future research endeavors can contribute to a more nuanced comprehension of employability skills' dynamic nature, the evolving demands of the job market, and the strategies needed to bridge the gap between education and employment.

## Data availability statement

Data included in article/supp. Material/referenced in article.

## CRediT authorship contribution statement

**Hasanuzzaman Tushar:** Conceptualization, Data curation, Formal analysis, Investigation, Visualization, Writing – original draft, Writing – review & editing. **Nanta Sooraksa:** Conceptualization, Methodology, Validation, Writing – review & editing.

## Declaration of competing interest

The authors declare that they have no known competing financial interests or personal relationships that could have appeared to influence the work reported in this paper.

## Figures and Tables

**Table:** 

Commonly reported skills	1990s	2000s	2010s	One time reported skills	1990s	2000s	2010s
1. Problem solving	5	5	12	1. Psychological Competency	0	0	1
2. Communication	4	6	11	2. Intellectual ability	0	1	0
3. Team Work	5	6	10	3. Work readiness	0	1	0
4. Adaptability	3	4	10	4. Career identity	0	1	0
5. Willingness to learn	4	3	10	5. Vitality	1	0	0
6. Creativity and initiative	4	5	8	6. Cooperation	1	0	0
7. ICT Skill	3	5	8	7. Self-promotion	1	0	0
8. Analytical/critical thinking	3	3	9	8. political awareness	1	0	0
9. Integrity	3	3	9	9. Commonsense	0	1	0
10. Interpersonal Skill	3	3	8	10. Sense of humor	0	1	0
11. Self-esteem	5	5	3	11. Ability to deal with pressure	0	1	0
12. Leadership	3	1	8	12. Sharing information	0	1	0
13. Planning and Organizing	3	4	5	13. Coaching	0	1	0
14. Self-Management	2	4	4	14. Mentoring and giving feedback	0	1	0
15. Responsibility	3	3	5	15. Generating opportunities	0	1	0
16. Positive Attitude and Behavior	1	3	6	16. Resourceful	0	1	0
17. Motivation	2	3	4	17. Personal developmental skill	0	1	0
18. Time management	0	4	4	18. Self-efficacy	0	1	0
19. Information management	2	3	3	19. Resilience	0	1	0
20. Basic literacy and numerical skill	2	3	2	20. Physical Fitness	0	0	1
21. Decision making	2	1	4	21. Safety	0	0	1
22. Professionalism	1	1	4	22. Accounting Skill	0	0	1
23. Career planning	1	1	4	23. Secretarial Skill	0	0	1
24. Sociability	1	2	3	24. Patience	0	0	1
25. Negotiation	2	2	1	25. Career advancement	0	0	1
26. Appearance and personal presentation	0	2	3	26. Technology design and programming	0	0	1
27. Academic Skill	1	1	3	27. Systems analysis and evaluation	0	0	1
28. Networking	1	2	2	28. Visionary thinking	1	0	0
29. Goal Setting	3	1	1	29. Quick responsiveness	0	0	1
30. Hard working	0	0	5	30. System Design	0	0	1
31. Reliability	0	2	3	31. Experiments/data analysis	0	0	1
32. Self-awareness	1	0	4	32. Kaizen Method	0	0	1
33. English Language Communication	0	2	4	33. Continuous improvement process	0	1	0
34. customer service	0	2	3				
35. Entrepreneurial skills	0	0	4				
36. Work knowledge	0	1	3				
37. Emotional intelligence	0	0	3				
38. Personality	1	1	1				
39. Work Independently	1	1	2				
40. Commitment	0	1	2				
41. Influencing Skill	0	0	2				
42. Self-discipline	0	0	3				
43. Technical skill	0	0	3				
44. Practical Experience	0	1	1				
45. Application of Skill	1	1	0				
46. Loyalty	0	1	1				
47. Enthusiasm/Passion	0	2	1				
48. Respecting others	0	1	1				
49. Commercial awareness	0	3	1				
50. Judgment Ability	0	0	2				
51. Selling Skill	0	0	2				
52. Use of modern tools	0	0	2				
53. Application of academic knowledge	0	0	2				
54. Dedication	1	0	1				
